# Supplementary material for: Do Preparation Techniques Transform the Metabolite Profile of Platelet-Rich Plasma?
Source: Bioengineering (Basel). 2025 Jul 17;12(7):774. doi: 10.3390/bioengineering12070774 (PMC12293010; doi:10.3390/bioengineering12070774)
Supplement: Supplementary file 1 [file bioengineering-12-00774-s001.zip › bioengineering-3694213-supplementary.pdf]

Table S1. Whole blood count before PRP preparations.

Variables	Mean ±SD	Unit
White blood cells	7.02±1.08	$10^3/\mu L$
Red blood cells	5.82±0.41	$10^6/\mu L$
Hemoglobin	16.57±1.27	g/dL
Hematocrit	48.60±3.90	%
Mean cell volume	83.58±3.83	fL
Mean cell hemoglobin	28.48±0.90	pg
Mean cell hemoglobin concentration	34.10±0.94	g/dL
Platelets	246.50±39.95	$10^3/\mu L$
Red Cell Distribution Width -SD	39.02±3.63	fL
Red Cell Distribution Width-Coefficient of Variation	12.87±1.17	%
Platelet Distribution Width	12.93±1.90	fL
Mean Platelet Volume	10.62±0.81	fL
Platelet Large Cell Ratio	31.03±7.64	%
PCT (Total thrombocyte volume)	0.26±0.05	%
Nucleated Red Blood Cell count Number #	0.00±0.00	$10^3/\mu L$
Nucleated Red Blood Cell count %	0.02±0.04	%
Neutrophils Number #	4.21±0.95	$10^3/\mu L$
Lymphocytes Number #	2.22±0.52	$10^3/\mu L$
Monocytes Number #	0.45±0.07	$10^3/\mu L$
Eosinophils Number #	0.11±0.11	$10^3/\mu L$
Basophils Number #	0.04±0.02	$10^3/\mu L$
Neutrophils %	59.57±7.80	%
Lymphocytes %	31.82±6.16	%
Monocytes %	6.38±0.84	%
Eosinophils %	1.67±2.10	%
Basophils %	0.57±0.25	%
Immunoglobulin Number #	0.03±0.02	$10^3/\mu L$
Immunoglobulin %	0.35±0.21	%
